# Copolymerization of CO_2_ and epoxides mediated by zinc organyls†

**DOI:** 10.1039/c7ra12535f

**Published:** 2018-01-18

**Authors:** Christoph Wulf, Ulrike Doering, Thomas Werner

**Affiliations:** Leibniz-Institut für Katalyse e. V. an der Universität Rostock Albert-Einstein-Str. 29a 18059 Rostock Germany Thomas.Werner@catalysis.de

## Abstract

Herein we report the copolymerization of CHO with CO_2_ in the presence of various zinc compounds R_2_Zn (R = Et, Bu, *i*Pr, Cy and Ph). Several zinc organyls proved to be efficient catalysts for this reaction in the absence of water and co-catalyst. Notably, readily available Bu_2_Zn reached a TON up to 269 and an initial TOF up to 91 h^−1^. The effect of various parameters on the reaction outcome has been investigated. Poly(ether)carbonates with molecular weights up to 79.3 kg mol^−1^ and a CO_2_ content of up to 97% were obtained. Under standard reaction conditions (100 °C, 2.0 MPa, 16 h) the influence of commonly employed co-catalysts such as PPNCl and TBAB has been investigated in the presence of Et_2_Zn (0.5 mol%). The reaction of other epoxides (*e.g.* propylene and styrene oxide) under these conditions led to no significant conversion or to the formation of the respective cyclic carbonate as the main product.

## Abstract 2

Simple zinc organyls (R_2_Zn) efficiently catalyze the copolymerization of CO_2_ and cyclohexene oxide. The effect of various reaction parameters has been studied. The reaction proceeds under halogen-free conditions and no co-catalyst is required.

## Graphical abstract

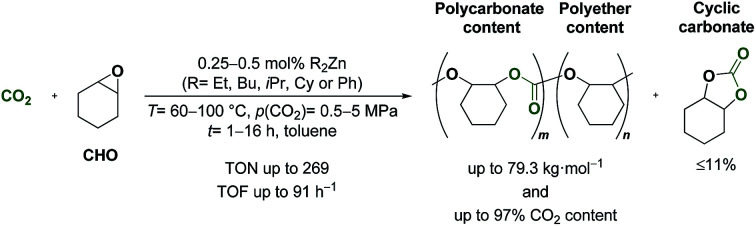

## 1. Introduction

**Scheme 1:**
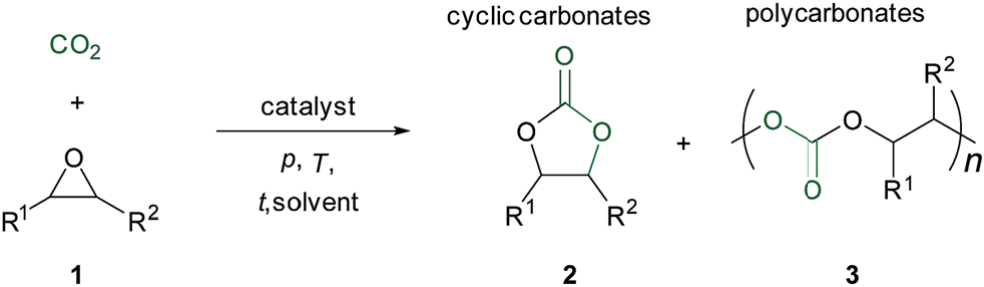
Reaction of epoxides and CO_2_.

CO_2_ is the by-product from combustion of fossil resources and chemical processes and its increasing concentration in the atmosphere is linked to global climate change.^1^ Thus, the utilization of the greenhouse gas CO_2_ as a C1-building block^2–4^ has attracted much attention due to its low cost, availability and the potential to substitute fossil fuel based feedstocks. Reductive transformations of CO_2_ to produce basic chemicals, *e.g.* formic acid^5^ or methanol require stoichiometric amounts of reductants such as silanes, boranes or hydrogen.^6^ In contrast the addition of CO_2_ to strained rings, such as oxetanes or epoxides, is a non-reductive process.^7^ The catalytic coupling of CO_2_ with epoxides 1 to generate cyclic carbonates 2 or polycarbonates 3 is an atom economic reaction (Scheme 1). The thermodynamically favored product of this reaction is the cyclic carbonate 2.^8^ Lower reaction temperatures and suitable catalysts allow kinetic control, thus the polycarbonates might be favored.

Over the past two decades significant efforts have been made in industry and academia to develop efficient catalysts for the selective formation of either cyclic carbonates^9–12^ or polycarbonates from epoxides and CO_2_.^13–18^

Polycarbonates produced by this reaction are used even on industrial scale *e.g.* for the production of polyurethanes.^19–23^ Current industrial processes for the production of polycarbonates are based on the condensation of diols with highly toxic phosgene. In 1969 Inoue and co-workers were the first to report the synthesis of poly(propylene carbonate) from CO_2_ and propylene oxide utilizing partially hydrolyzed Et_2_Zn to initiate the polymerization.^24,25^ Since this pioneering work many catalysts have been developed for the copolymerization of CO_2_ with epoxides.^13–18^ Especially zinc based catalyst systems were shown to be efficient. A variety of initiating systems based on different zinc species alone^26,27^ as well as in combination with additives *e.g.* ZnO and diprotic activators (*e.g.* glutaric acid)^28^ have been reported.^29–31^ Moreover, transition metal complexes based on zinc proved to be highly efficient and selective. In this context, Darensbourg *et al.* developed zinc phenoxide catalysts, which exhibit high turnover capabilities for the copolymerization of cyclohexene oxide (CHO) and CO_2_.^32–34^ Coates and co-workers developed zinc-β-diiminate-complexes for the synthesis of monodispersed, highly alternating copolymers with high molecular weight.^35–38^ The group of Williams demonstrated a zinc-based macrocyclic bimetallic catalyst, which showed remarkable activity even at atmospheric pressure of carbon dioxide.^39–41^ More recently, Rieger *et al.*^42–44^ reported dinuclear zinc-β-diiminate complexes and Dinjus and co-workers^45^ complexes based on the N_4_–*N*,*N*-bis(2-pyridinecarboxamide)-1,2-benzene chelating ligand for the copolymerization of CO_2_ and CHO.

Notably, most of these systems require halogen containing compounds, such as ammonium and phosphonium salts which are commonly employed co-catalyst for the synthesis of cyclic- as well as polycarbonates from epoxides and CO_2_. We are generally interested in the reaction between epoxides and CO_2_.^46–54^ Most recently we reported a zinc based binary catalytic system for the synthesis of cyclic carbonates.^55^ Herein we report the efficient copolymerization of CHO and CO_2_ in the presence of organozinc compounds in catalytic amounts under co-catalyst and halogen-free conditions.

## 2. Experimental

### 2.1 Material

The zinc organyls ZnR_2_ were obtained in solution from commercial sources as follows and used as received: Et_2_Zn (*c* = 1.1 M in toluene) from Sigma Aldrich, Bu_2_Zn (*c* = 1.0 M in heptane) from Acros, *i*Pr_2_Zn (*c* = 1.0 M in toluene) from Sigma Aldrich, Cy_2_Zn (*c* = 0.4 M in diethyl ether) from Sigma Aldrich and Ph_2_Zn as solid from Strem. Epoxides were obtained from commercial sources as follows and distilled over CaH_2_ prior the use: cyclohexene oxide from Acros, propylene oxide from Acros, styrene oxide from Acros, 4-methyl-1,2-cyclohexene oxide from Alfa Aesar, (+)-limonene-1,2-epoxide and butylene oxide from Sigma Aldrich and 2-(3,4-epoxycyclohexyl)-ethyl-trimethoxy-silane and 2-(3,4-epoxycyclohexyl)-ethyl-triethoxy-silane from abcr. All solvents were obtained dried from Acros over molecular sieves.

### 2.2 Measurements

^1^H and ^13^C spectra were recorded with a Bruker 300 Fourier (300 MHz), Bruker AV 300 (300 MHz) and Bruker AV 400 (400 MHz). Shifts *δ* are stated in ppm. The spectra were calibrated to the rest signal of the applied solvent. CDCl_3_: ^1^H *δ* = 7.27 ppm, ^13^C *δ* = 77.00 ppm.

The experiments were carried out under increased pressure in a Multiple Reactor System 5000 and Compact Micro Reactor 5000 from Parr. The molar masses and dispersities were analyzed employing size exclusion chromatography (SEC) 1100 GPC from Agilent Technologies with a refraction index detector at 40 °C. The measurements were performed at a constant temperature of 40 °C using three columns with a polyester copolymer network as stationary phase (PSS GRAM 1000 Å, 5 μm particle size, 8.0 × 300 mm; PSS GRAM 100 000 Å, 5 μm particle size, 8.0 × 300 mm; PSS GRAM 1 000 000 Å). For calibration polystyrene standards from Polymer Standards Service (PSS) were used. Unstabilized THF (HPLC grade) was applied as the mobile phase with a flow rate of 1 mL min^−1^. For this purpose around 10 mg of the sample were dissolved in 1 mL THF. Ethylene glycol was used as reference peak. For the recording and the evaluation of the measurement the software PSS WINGPC 6® UniChrom from PSS was used.

### 2.3 General procedures for the copolymerization with zinc organyls

In a 45 cm^3^ stainless-steel autoclave a solution of R_2_Zn (0.25 mmol, 0.5 mol%) was added dropwise to a solution of cyclohexene oxide (4.91 g, 50 mmol) in 2 mL toluene. The reactor was sealed and charged with 0.5–5.0 MPa CO_2_ at 60–100 °C. The reaction mixture was stirred for 1–48 h. Subsequently, the reactor was cooled to ≤20 °C in an ice bath and CO_2_ was released slowly. After the removal of all volatile components in a vacuum the polymer was solved in 20 mL of CH_2_Cl_2_ and precipitated with 50 mL of a solution of MeOH and 5% HCl. The precipitate was filtered off and dried to yield a polymer as a colorless solid.

### 2.4 General procedure for the copolymerization with Et_2_Zn

In a 45 cm^3^ stainless-steel autoclave Et_2_Zn (0.25 mmol, 0.23 mL, 15 wt% in toluene, 0.5 mol%) was added dropwise to a solution of cyclohexene oxide (4.91 g, 50 mmol) in 2 mL toluene. The reactor was sealed and charged with 2.0 MPa CO_2_ at 100 °C. The reaction mixture was stirred for 16 h. Subsequently, the reactor was cooled to ≤20 °C in an ice bath and CO_2_ was released slowly. After the removal of all volatile components in vacuum the polymer was solved in 20 mL of CH_2_Cl_2_ and precipitated with 50 mL of a solution of MeOH and 5% HCl. The precipitate was filtered off and dried to yield 5.28 g polymer (66%) as a colorless solid.

## 3. Results and discussion

Initially we studied different readily available organozinc compounds as catalysts under standard reaction conditions (0.5 mol% R_2_Zn, 100 °C, 2.0 MPa, 16 h, Table 1). In the absence of R_2_Zn neither the formation of polycarbonate nor cyclohexene carbonate (CHC) was observed (entry 1). In the presence of Et_2_Zn (0.5 mol%) the formation of a polycarbonate with a carbonate content of >85% was obtained as the main product (entry 2). Interestingly, this was achieved in the absence of a co-catalyst and without the addition of water. Even though the use of Bu_2_Zn led to a product with higher molecular weight the incorporation of CO_2_ decreased significantly <80% (entry 3). *i*Pr_2_Zn and Cy_2_Zn gave similar results in respect to the TON and polycarbonate content while the *M*_n_ of the product almost doubled in the presence of Cy_2_Zn (entry 4 and 5). The polymer with the lowest *M*_n_ of 11.7 kg mol^−1^ and a polycarbonate to polyether ratio of 81 : 19 was obtained with Ph_2_Zn (entry 6). As expected the reduction of the amount of Et_2_Zn from 0.5 mol% to 0.25 mol% led to a significant increase of the *M*_n_ from 10.5 to 43.1 kg mol^−1^ respectively (entry 2 *vs.* 7). Even though the CO_2_-content also increased to >99%, the yield dropped below 10%. Similar trends were observed when 0.25 mol% of R_2_Zn (R = Bu, *i*Pr, Cy and Ph) were employed.^47,56^ Notably, if the reaction was carried out under argon instead of CO_2_ a high molecular polyether (*M*_n_ = 76.4 kg mol^−1^) was obtained in 16% yield (entry 8). Even though the highest yields were obtained in toluene other solvents such as CH_2_Cl_2_ and THF are also suitable.^47,56^ For further investigations we did MALDI-TOF experiments for the polymer obtained with Ph_2_Zn (Table 1, entry 6). The spectrum shows the alternating nature of the polycarbonate with repeating units for the monomer of 142 g mol^−1^ (Fig. S1†).

**Table 1:** Screening of readily available zinc organyls R_2_Zn for the copolymerization of CHO and CO_2_^a^

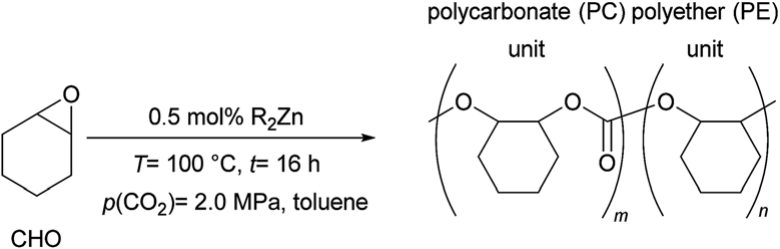							
Entry	R_2_Zn	TON	*Y* b ^,^ c/%	PC : PE ratio^d^	*M*_n_/kg mol^−1^^e^	*Đ* e	*T*_g_/°C
1	—	—	0 (0)	—	—	—	—
2	Et_2_Zn	145	63 (7)	87 : 13	10.5	5.0	89.5
3	Bu_2_Zn	154	60 (10)	77 : 23	20.8	3.6	98.1
4	*i*Pr_2_Zn	166	64 (9)	87 : 13	12.1	3.0	—
5	Cy_2_Zn	162	66 (11)	90 : 10	22.2	2.6	—
6	Ph_2_Zn	107	46 (7)	81 : 19	11.7	4.4	81.6
7^f^	Et_2_Zn	65	6 (<1)	>99 : <1	43.1	4.6	83.1
8^g^	Et_2_Zn	31	16 (0)	<1 : >99	76.4	2.2	—

a Reaction conditions: 50 mmol CHO, 0.5 mol% R_2_Zn, 2 mL toluene, *p*(CO_2_) = 2.0 MPa, *T* = 100 °C, *t* = 16 h.

b Isolated yield of the polymer after precipitation from CH_2_Cl_2_ with 5 mol% HCl in MeOH.

c Yield of CHC determined by ^1^H NMR from the reaction mixture in parenthesis.

d Determined by comparison of the integrals arising from the methine protons in the ^1^H NMR spectra from the polycarbonate (PC) and polyether (PE) unit.

e Determined by SEC in THF, calibrated with polystyrene standards.

f 0.25 mol% Et_2_Zn.

g Reaction was carried out under inert atmosphere (argon).

Subsequently, we studied the influence of various reaction parameters (*p*(CO_2_), *T*, *t*) for all of the tested zinc compounds since all of them showed similar activity and selectivity as well as promising results in respect to the polymer properties such as amount of incorporated CO_2_ and *M*_n_.

First the influence of the CO_2_-pressure on the copolymerization of CHO and CO_2_ was investigated (Table 2). For all of the tested zinc compounds R_2_Zn an increase of the pressure to 5.0 MPa led to higher turnover numbers and increased isolated polymer yields compared to 2.0 MPa (Table 2, entries 1–5 *vs.*Table 1, entries 2–6).

**Table 2:** Influence of the CO_2_-pressure on the copolymerization of CHO and CO_2_ in the presence of various zinc organyls R_2_Zn^a^

Entry	R_2_Zn	*p*(CO_2_)/MPa	TON	*Y* b ^,^ c/%	PC : PE ratio^d^	*M*_n_/kg mol^−1^^e^	*Đ* e
1	Et_2_Zn	5	167	67 (5)	94 : 6	16.8	5.5
2	Bu_2_Zn	5	197	65 (7)	68 : 32	24.9	3.1
3	*i*Pr_2_Zn	5	174	87 (4)	90 : 10	11.4	5.7
4	Cy_2_Zn	5	170	51 (7)	97 : 3	21.8	3.0
5	Ph_2_Zn	5	137	59 (8)	87 : 13	22.8	3.2
6	Et_2_Zn	0.5	70	20 (3)	81 : 19	9.9	6.3
7	Bu_2_Zn	0.5	79	18 (4)	29 : 71	11.6	4.6
8	*i*Pr_2_Zn	0.5	100	33 (5)	68 : 32	7.7	5.3
9	Cy_2_Zn	0.5	54	18 (4)	81 : 19	4.3	4.4
10	Ph_2_Zn	0.5	57	15 (2)	29 : 71	6.9	3.8

a Reaction conditions: 50 mmol CHO, 0.5 mol% R_2_Zn, 2 mL toluene, *p*(CO_2_) = 0.5–5.0 MPa, *T* = 100 °C, *t* = 16 h.

b Isolated yield of the polymer after precipitation from CH_2_Cl_2_ with 5 mol% HCl in MeOH.

c Yield of CHC determined by ^1^H NMR from the reaction mixture in parenthesis.

d Determined by comparison of the integrals arising from the methine protons in the ^1^H NMR spectra from the PC and PE unit.

e Determined by SEC in THF, calibrated with polystyrene standards.

Interestingly, in the presence of Et_2_Zn the *M*_n_ increased from 10.5. to 16.8 kg mol^−1^ while for Ph_2_Zn the *M*_n_ increased to 22.8 kg mol^−1^ (Table 2, entries 1 and 5 *vs.*Table 1, entries 2 and 6). At a lower CO_2_ pressure of 0.5 MPa the turnover number, polymer yield and polycarbonate content decreased for all R_2_Zn compounds (Table 2, entries 6–10 *vs.*Table 1, entries 2–6). Except for Et_2_Zn the *M*_n_ dropped significantly.

Since the polycarbonate is the kinetic product of the reaction of CO_2_ and CHO, we subsequently studied the influence of the reaction temperature.^8^ Thus the reaction was carried out at 60 °C in the presence of the different organozinc compounds (Table 3). As expected in all cases the formation of the cyclic carbonate which is the thermodynamic product decreased significantly for the different ZnR_2_ while the turnover number decreased slightly in all cases (Table 3*vs.*Table 1, entries 2–6). Notably, for Et_2_Zn the polycarbonate content dropped from 94% to 16% while the *M*_n_ increased from 10.5 to 79.3 kg mol^−1^ (Table 3, entry 1 *vs.*Table 1, entry 2). In the presence of the other zinc compounds ZnR_2_ (R = Bu, *i*Pr, Cy or Ph) polymers obtained at 60 °C showed similar CO_2_ content compared the products which were obtained at 100 °C (Table 3, entries 2–5 *vs.*Table 1, entries 3–6). Interestingly, for *i*Pr_2_Zn and Ph_2_Zn the *M*_n_ at lower temperature was about three times higher compared to 100 °C (Table 3, entries 3 and 5 *vs.*Table 1, entries 4 and 6). The observation that with decreasing reaction temperature the *M*_n_ increases is in accordance with work previously reported by Soga and co-workers.^56^ They investigated the effect of the temperature on the copolymerization of propylene oxide and CO_2_ using an alumina-supported diethylzinc catalyst. In general the observed dispersities of the products were higher compared to the polymers obtained at 100 °C (Table 3, entries 1–5 *vs.*Table 1, entries 2–6).

**Table 3:** Influence of the temperature on the copolymerization of CHO and CO_2_^a^

Entry	R_2_Zn	*T*/°C	TON	*Y* b ^,^ c/%	PC : PE ratio^d^	*M*_n_/kg mol^−1^^e^	*Đ* e
1	Et_2_Zn	60	113	52 (0)	16 : 84	79.3	4.4
2	Bu_2_Zn	60	123	53 (0)	71 : 29	23.0	10.3
3	*i*Pr_2_Zn	60	115	45 (0)	84 : 16	37.5	7.3
4	Cy_2_Zn	60	80	52 (0)	94 : 6	27.9	7.4
5	Ph_2_Zn	60	91	37 (≤1)	68 : 32	30.2	7.3

a Reaction conditions: 50 mmol CHO, 0.5 mol% R_2_Zn, 2 mL toluene, *p*(CO_2_) = 2.0 MPa, *T* = 60 °C, *t* = 16 h.

b Isolated yield of the polymer after precipitation from CH_2_Cl_2_ with 5 mol% HCl in MeOH.

c Yield of CHC determined by ^1^H NMR from the reaction mixture in parenthesis.

d Determined by comparison of the integrals arising from the methine protons in the ^1^H NMR spectra from the PC and PE unit.

e Determined by SEC in THF, calibrated with polystyrene standards.

**Fig. 1:**
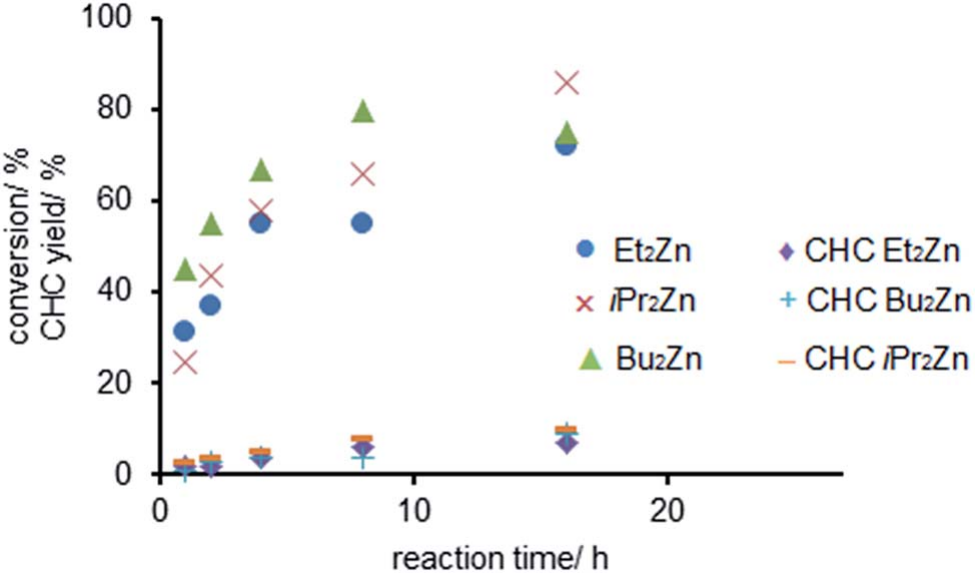
Influence of the dialkyl zinc compounds (ZnR_2_, R = Et, Bu, *i*Pr) on the conversion of CHO (copolymerization of CHO and CO_2_, observed CHC ≤10%) in dependence on the reaction time. Reaction conditions: 50 mmol CHO, 0.5 mol% R_2_Zn, 2 mL toluene, *p*(CO_2_) = 2.0 MPa, *T* = 100 °C, *t* = 1–16 h. The data points are the results for separate batches.

Consequently we investigated the influence of the reaction time on the copolymerization for three different dialkyl zinc compounds (R_2_Zn, R = Et, Bu, *i*Pr). Bu_2_Zn showed the highest initial activity with 45% conversion of the starting material after 1 h (Fig. 1).^47,56^ In the presence of Et_2_Zn and *i*Pr_2_Zn the conversion of CHO was 31% and 25% after 1 h, respectively. Notably, after 4 h more than 50% of the starting material was converted. Subsequently the reaction rate decreased in all cases which resulted in a maximum conversion of only 86% for *i*Pr_2_Zn after 16 h. The exponential formation of the polycarbonate was accompanied by a linear increase in the cyclic carbonate content of the reaction mixture which is in accordance with previously reported work by Górecki and Kuran.^47,57^ Notably, the CHC-content in all cases did not exceed 10% after 16 h.

**Fig. 2:**
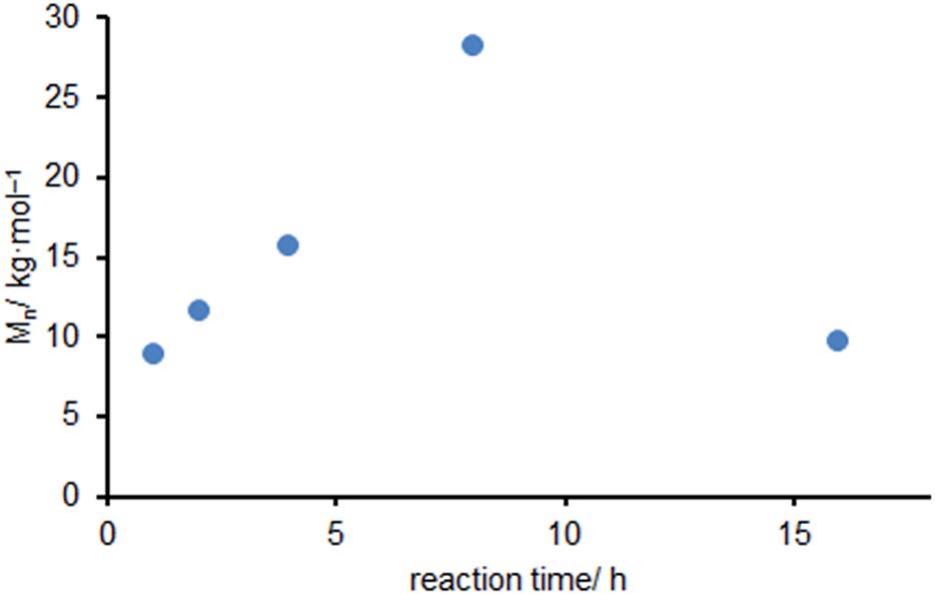
Influence of the dialkyl zinc compound Et_2_Zn on the *M*_n_ of CHO (copolymerization of CHO and CO_2_, observed CHC ≤10%) in dependence on the reaction time. Reaction conditions: 50 mmol CHO, 0.5 mol% R_2_Zn, 2 mL toluene, *p*(CO_2_) = 2.0 MPa, *T* = 100 °C, *t* = 1–16 h. The data points are the results for separate batches.

In the presence of Et_2_Zn the *M*_n_ of the obtained polycarbonate showed a linear increase from 8.9 to 28.3 kg mol^−1^ during the first 8 h (Fig. 2). Subsequently the *M*_n_ decreased to 12.2 kg mol^−1^ after 16 h. This might be addressed to transesterification reactions as suggested by Vandenberg and Tian.^58^ This result is comparable to the molecular weights obtained by Williams *et al.* using mixed salts prepared from Et_2_Zn and acetic acid as a catalyst.^59^

Notably, no clear trend was observed for the other zinc compounds (R_2_Zn, R = Bu and *i*Pr).^47,56^

However, in all cases the CO_2_ content of the obtained polymer was approximately constant (85%) over time. In further investigations, the influence of different co-catalysts was tested (Table 4). The copolymerization was performed with Et_2_Zn (0.5 mol%) under standard reaction conditions (2.0 MPa, 100 °C, 16 h) in the presence of commonly used co-catalysts.^60–66^ Notably, in all cases the utilization of a co-catalyst led only to the formation of the cyclic carbonate and/or low molecular weight products/oligomers. Tetra-*n*-butylammonium bromide (TBAB) and bis(triphenylphosphine)iminium chloride (PPNCl) are known to catalyze the addition of CO_2_ to epoxides yielding cyclic carbonates.^67,68^ Both co-catalyst provided similar results mainly producing the cyclic carbonate in 63% and 82% yield respectively (entry 1 and 2). Known as catalysts for ring-opening-polymerization (ROP) of cyclic carbonates, 1,8-diazabicyclo(5.4.0)undec-7-ene (DBU) and triazabicyclodecene (TBD) were used as co-catalyst to decrease the cyclic side product.^64,69^ In the presence of DBU the formation of an oligomer (*M*_n_ = 510 g mol^−1^) with a polycarbonate content of 55% was observed (entry 3). A similar result was obtained with TBD as the co-catalyst (entry 4) while 4-dimethylaminopyridine (DMAP) showed no significant conversion (entry 5). It is generally discussed that TBD hinders the depolymerization of the polycarbonate through deprotonation of the *in situ* formed hydroxyl end-group.^70,71^

**Table 4:** Evaluation of co-catalysts for the Et_2_Zn mediated copolymerization of CHO with CO_2_^a^

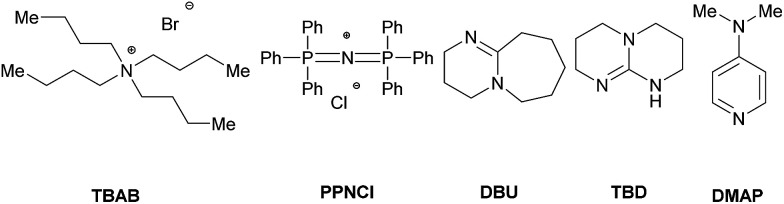					
Entry	Co-catalyst	TON	*Y* b ^,^ c/%	PC : PE ratio^d^	*M*_n_/mol^−1^^e^
1	TBAB	141	7 (63)	0 : 100	270
2	PPNCl	172	3 (82)	0 : 100	260
3	DBU	66	18 (14)	55 : 45	510
4	TBD	61	18 (12)	71 : 29	590
5	DMAP	16	8 (<1)	65 : 35	490

a Reaction conditions: 50 mmol CHO, 0.5 mol% R_2_Zn, 0.5 mol% co-catalyst, 2 mL toluene, *p*(CO_2_) = 2.0 MPa, *T* = 100 °C, *t* = 16 h.

b Yield of polymer determined by ^1^H NMR from the reaction mixture.

c Yield of CHC determined by ^1^H NMR from the reaction mixture in parenthesis.

d Determined by comparison of the integrals arising from the methine protons in the ^1^H NMR spectra from the PC and PE unit.

e Determined by SEC in THF, calibrated with polystyrene standards.

Additionally, we studied the conversion of various other epoxides with CO_2_. In the presence of Et_2_Zn (0.5 mol%) under our standard reaction conditions (2.0 MPa, *T* = 100 °C, *t* = 16 h, Table 5). For the simple propylene oxide a conversion of 8% and low weight polymer with high polyether content was reached (entry 1). This is a lower polymer yield then previous reported by Inoue where 5.0–6.0 MPa CO_2_-pressure and the Et_2_Zn/H_2_O system was used.^24,25^ For the conversion of butylene oxide only 7% of the oligomeric ether and 5% of the respective cyclic carbonate were observed (entry 2). The reaction of styrene oxide led to higher TON of 59 and 20% yield of an oligomer with a *M*_n_ < 500 g mol^−1^ (entry 3) which is higher than the obtained yield of 2% by the Et_2_Zn/H_2_O system by Endo and co-workers. Epichlorohydrin showed a poor yield of the corresponding cyclic carbonate of only 2% (entry 4) which is lower than the result of Endo and co-workers.^24^ Notably, other cyclohexane based epoxides could not be converted to the corresponding polycarbonates (entries 5–8).

**Table 5:** Evaluation of different epoxides^a^

Entry	Monomer	*X*_epoxide_/%	TON	*Y* b ^,^ c/%	*M*_n_/g mol^−1^^d^
1^e^	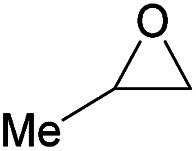	8	15	7^j^ (<1)	<500
2^f^	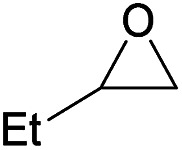	12	25	7^j^ (5)	<500
3^e^	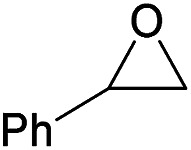	28	59	20^j^ (9)	<500
4^f^	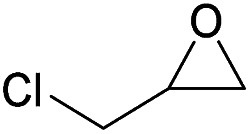	2	1	0 (2)	—
5^f^	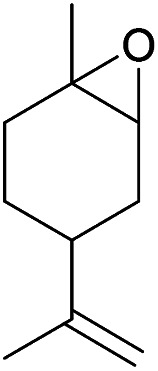	<1	—	—	—
6^g^	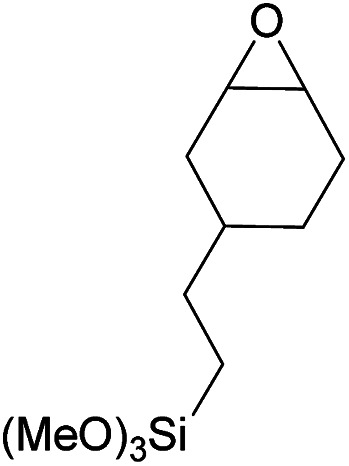	<1	—	—	—
7^h^	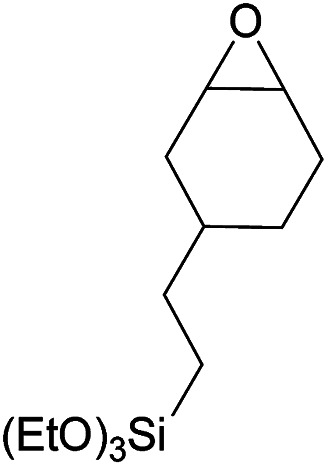	<1	—	—	—
8^i^	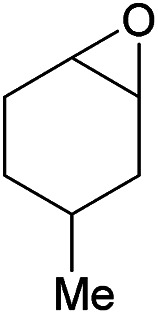	27	57	27^j^ (0)	<500

a Reaction conditions: 0.5 mol% Et_2_Zn, 2 mL toluene, p(CO_2_) = 2.0 MPa, *T* = 100 °C, *t* = 16 h.

b Yield of polymer determined by ^1^H NMR from the reaction mixture.

c Yield of CHC determined by ^1^H NMR from the reaction mixture in parenthesis.

d Determined by SEC in THF, calibrated with polystyrene standards.

e 50 mmol epoxide.

f 25 mmol epoxide.

g 17 mmol epoxide.

h 14 mmol epoxide.

i 11 mmol epoxide.

j Only Polyether was observed.

In the case of limonene oxide this might be addressed to steric hindrance which was observed before by Coates^71^ and Anwander^72^ (entry 5). The alkoxy silyl functionalized substrates showed no conversion which might be addressed to a reaction of these groups with the catalyst.^72,73^ The methyl substituted cyclohexene oxide was partially converted to an oligomeric ether (entry 8).

## 4. Conclusions

Zinc organyls (R_2_Zn, R = Et, Bu, *i*Pr, Cy and Ph) efficiently mediate the copolymerization of CO_2_ and CHO. Under the standard reaction conditions (100 °C, 2.0 MPa) an initial TOF of up to 91 h^−1^ (for Bu_2_Zn) and TONs up to 269 after 16 h were achieved. Polycarbonates with molecular weights up to 79.3 kg mol^−1^ and a CO_2_ content up to 97% were obtained. The effect of various parameters on the reaction outcome has been investigated. An increase of the pressure to 5.0 MPa led to higher turnover numbers and increased isolated polymer yields compared to 2.0 MPa. However, at this pressure also the highest dispersities were observed. Higher molecular weight products could be isolated at lower reaction temperature of 60 °C. Several commonly employed readily available co-catalysts were studied in combination with Et_2_Zn. However, the co-catalysts facilitated rather the formation of the cyclic carbonate than the production of the polycarbonate. Moreover different epoxides were tested in the copolymerization with CO_2_, unfortunately only low conversions and/or the formation of the respective cyclic carbonates were observed as the major product.

## Conflicts of interest

There are no conflicts to declare.

## Supplementary Material

RA-008-C7RA12535F-s001
