# Supplementary material for: Copolymerization of CO2 and epoxides mediated by zinc organyls
Source: RSC Adv. 2018 Jan 18;8(7):3673–9. doi: 10.1039/c7ra12535f (PMC9077689; doi:10.1039/c7ra12535f)
Supplement: RA-008-C7RA12535F-s001 [file RA-008-C7RA12535F-s001.pdf]

Electronic Supplementary Material (ESI) for RSC Advances.
This journal is © The Royal Society of Chemistry 2017

SUPPORTING INFORMATION

Copolymerization of CO_2 and Epoxides mediated by zinc organyls

Christoph Wulf,[a] Ulrike Doering,[a] Thomas Werner*[a]

[a] Leibniz-Institut für Katalyse e. V. an der Universität Rostock

Albert-Einstein-Str. 29a, 18059 Rostock, Germany. E-mail: Thomas.Werner@catalysis.de

Table of Content

Table S1. Optimizing catalyst amount of Zinc organyls.*a* S1

Table S2. Influence of the solvents and volume-ratios on the copolymerization.*a* S1

Table S3. Influence of the reaction time for the copolymerization of CHO and CO_2. .... S2

Figure S1. MALDI-TOF mass spectrum of polycarbonate obtained after optimized conditions (Table 1, entry 6): zoom of the region *m/z*= 1000–3500 *m/z*, picked peaks show multiples of monomer unit with *m/z*= 142. .... S3

Table S1. Optimizing catalyst amount of zinc organyls.[a]

Entry	R_2Zn	*TON*	$Y^{b,c}$/%	*PC:PE ratio*[d]	M_n/ kg·mol^{-1} [e]	$Đ^e$
1	Et_2Zn	65	6 (<1)	>99 : <1	43.1	4.6
2	Bu_2Zn	269	52 (3)	94 : 6	18.7	4.8
3	*i*Pr_2Zn	120	16 (<1)	90 : 10	15.8	5.6
4	Cy_2Zn	85	6 (<1)	97 : 3	22.5	5.2
5	Ph_2Zn	44	3 (<1)	94 : 6	33.1	2.4

[a] Reaction conditions: 50 mmol CHO, 0.25 mol% R_2Zn, 2 mL toluene, $p(CO_2)$= 2.0 MPa, T= 100°C, t= 16 h. [b] Isolated yield of the polymer after precipitation from CH_2Cl_2 with 5 mol% HCl in MeOH. [c] Yield of CHC determined by ^1H NMR from the reaction mixture in brackets. [d] Determined by comparison of the integrals arising from the methine protons in the ^1H NMR spectra from the PC and PE unit. [e] Determined by SEC in THF, calibrated with polystyrene standards.

Table S2. Influence of the solvents and volume-ratios on the copolymerization.[a]

Entry	*Solvent*	*TON*	$Y^{b,c}$/%	*PC:PE ratio*[d]	M_n/ kg·mol^{-1} [e]	$Đ^e$
1	-	27	21 (4)	87 : 13	30.8	3.4
2	THF	30	55 (1)	97 : 3	58.9	3.8
3	Toluol	30	73 (1)	97 : 3	31.5	4.4
4	DCM	29	53 (4)	94 : 6	25.6	4.5

[a] Reaction conditions: 25 mmol CHO, 0.25 mol% Et_2Zn, $V_{(solvent)}$= 1 mL, $p(CO_2)$= 3.8 MPa, T= 100°C, t= 16 h. [b] Isolated yield of the polymer after precipitation from CH_2Cl_2 with 5 mol% HCl in MeOH. [c] Yield of CHC determined by ^1H NMR from the reaction mixture in brackets. [d] Determined by comparison of the integrals arising from the methine protons in the ^1H NMR spectra from the PC and PE unit. [e] Determined by SEC in THF, calibrated with polystyrene standards.

Table S3. Influence of the reaction time for the copolymerization of CHO and CO_2.

Entry	R_2Zn	*t*/ h	X_{CHO}/ %	$Y_{Polymer}{}^{b,c}$/ %	$Y_{CHC}{}^{b}$/ %	CO_2 content/ %[d]	M_n/ $kg \cdot mol^{-1}$ [e,f]
1	Et_2Zn	1	31	29 (23)	2	71	8.9 (15.4)
2	Et_2Zn	2	37	34 (33)	2	90	11.5 (15.0)
3	Et_2Zn	4	55	51 (49)	4	90	15.8 (17.3)
4	Et_2Zn	8	55	50 (50)	6	90	28.3 (32.0)
5	Et_2Zn	16	73	66 (63)	7	87	9.7 (10.5)
6	Bu_2Zn	1	45	42 (36)	3	71	13.6 (13.6)
7	Bu_2Zn	2	55	51 (45)	4	87	7.9 (14.3)
8	Bu_2Zn	4	67	62 (57)	5	87	13.3 (15.2)
9	Bu_2Zn	8	80	72 (67)	8	84	10.9 (11.4)
10	Bu_2Zn	16	79	65 (60)	10	77	14.4 (20.8)
11	*i*Pr_2Zn	1	25	24 (20)	1	87	5.7 (8.8)
12	*i*Pr_2Zn	2	44	41 (38)	3	87	10.2 (11.9)
13	*i*Pr_2Zn	4	58	54 (50)	4	90	8.9 (14.5)
14	*i*Pr_2Zn	8	66	62 (56)	4	90	9.5 (10.3)
15	*i*Pr_2Zn	16	86	77 (64)	9	87	5.1 (12.1)

[a] Reaction conditions: 50 mmol CHO, 0.5 mol% R_2Zn, 2 mL toluene, $p(CO_2)$= 2.0 MPa, T= 100°C, t= 1–48 h. [b] Yield determined by ^1H NMR from the reaction mixture [c] Isolated yield of the polymer after precipitation from CH_2Cl_2 with 5 mol% HCl in MeOH in brackets. [d] Determined by comparison of the integrals arising from the methine protons in the ^1H NMR spectra from the PC and PE unit. [e] Determined by SEC in THF, calibrated with polystyrene standards. [f] M_n of the polymer after precipitation from CH_2Cl_2 with 5 mol% HCl in MeOH in brackets.

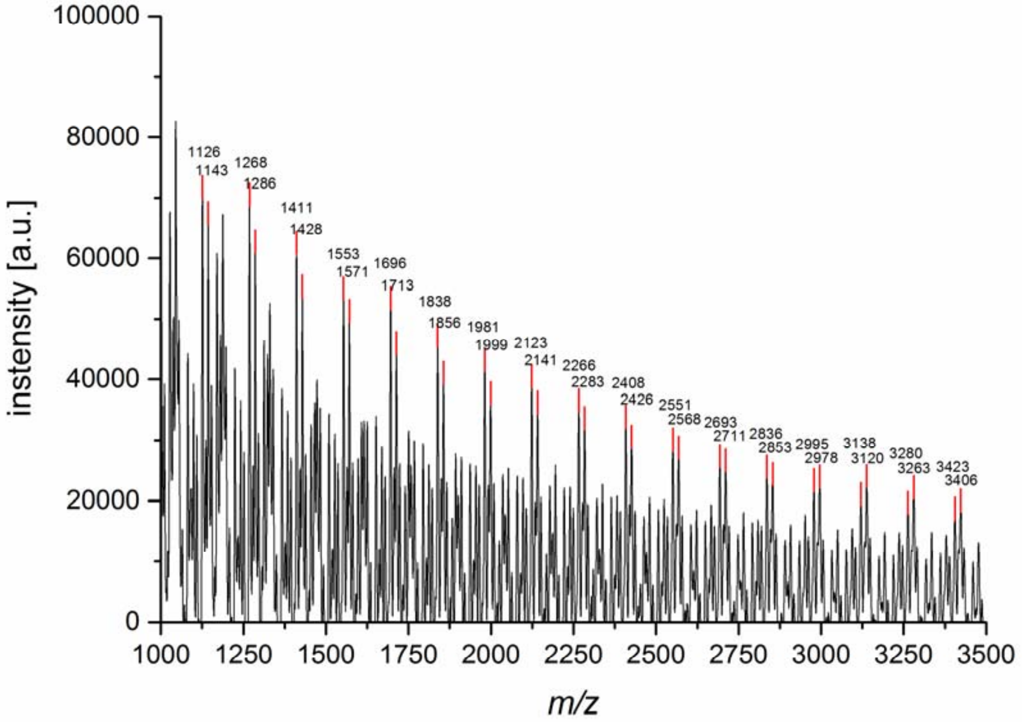

Figure S1. MALDI-TOF mass spectrum of polycarbonate obtained after optimized conditions (Table 1, entry 6): zoom of the region *m/z*= 1000–3500 *m/z*, picked peaks show multiples of monomer unit with *m/z*= 142.
